# Low BUB1 expression is an adverse prognostic marker in gastric adenocarcinoma

**DOI:** 10.18632/oncotarget.19357

**Published:** 2017-07-18

**Authors:** David Stahl, Martin Braun, Andrew J. Gentles, Philipp Lingohr, Adeline Walter, Glen Kristiansen, Ines Gütgemann

**Affiliations:** ^1^ Institute of Pathology, University Hospital Bonn, Bonn, Germany; ^2^ Center for Cancer Systems Biology (CCSB), Stanford University, Stanford, California, USA; ^3^ Department of General, Visceral, Thoracic and Vascular Surgery, University Hospital Bonn, Bonn, Germany; ^4^ Department of Gynecology and Obstetrics, University Hospital Bonn, Bonn, Germany

**Keywords:** BUB1, immunohistochemistry, biomarker, gastric adenocarcinoma, gastric cancer

## Abstract

Gastric adenocarcinomas are associated with a poor prognosis due to the fact that the tumor has often metastasized by the time of diagnosis and prognostic markers are urgently needed to tailor treatment.

We examined the expression of the mitotic spindle checkpoint protein BUB1 (budding uninhibited by benzimidazoles 1) and Ki-67 protein expression by immunohistochemistry in 218 patients with primary gastric adenocarcinomas.

Tumors with low frequency of BUB1 expression were associated with larger tumor size (pT) (p < 0.001), higher incidence of lymph node metastases (pN) (p = 0.027), distant metastases (pM) (p = 0.006) and higher UICC stage (p < 0.001). Furthermore, BUB1 expression was inversely correlated with residual tumor stage (p = 0.038). Abundant BUB1 protein expression correlated with frequent Ki-67 protein expression (p < 0.001) and low BUB1 expression was associated with shorter survival (p < 0.001). Univariate and multivariate analyses confirmed BUB1 to be an independent prognostic marker in gastric cancer (p = 0.021).

## INTRODUCTION

Gastric cancer (GC) is the third leading cause for cancer related death after lung and liver cancer [1]. It is often diagnosed at an advanced stage when the primary tumor has already metastasized. Early stages are often clinically silent with nonspecific symptoms such as dyspepsia. However, early detection is important: if the tumor is detected and treated before it invades the muscular layer of the stomach the 5-year-survival rate is 90% in comparison to 5-year-survival at an advanced stage of <20%. Prognostic biomarkers in advanced disease are the most important instrument for tailoring treatment. Unfortunately, in GC, neither histologic subtype nor tumor stage according to the classification of the UICC is able to sufficiently predict prognosis [2, 3].

Classic biomarkers for GC diagnosis include carcinoembryonic antigen and cancer antigen 19-9 [4]; however, these are not always expressed, have small sensitivity and specificity and have no prognostic value.

Recently, the proliferative rate assessed by Ki-67 in tumor cells has gained increasing importance in prognostication and stratification of various cancers such as breast cancer [5], lymphoma and neuroendocrine neoplasia [6, 7], however, Ki-67 has shown to be of limited use in GC [8].

Budding uninhibited by benzimidazoles 1 (BUB1) is highly expressed during mitosis and correlates with cell proliferation [9]. Recently, BUB1 was reported to be expressed in GC [10] and this overexpression did not correlate with DNA ploidity or microsatellite instability [11].

BUB1 is a serine/threonine kinase protein bound to the kinetochore with its N-terminus. It is essential for spindle assembly checkpoint (SAC) signaling and for correct chromosome alignment during mitosis [12]. During chromosome segregation microtubules need to be aligned properly to the kinetochore pairs. The spindle checkpoint controls this process by cell cycle delay in metaphase. In healthy cells, incorrectly attached chromosomes lead to an inhibition of the anaphase promoting complex or cyclosome (APC/C) by formation of the mitotic checkpoint complex (MCC) and phosphorylation of CDC20, both inhibiting APC/C [13]. MCC consists of BUBR1, BUB3, MAD2 and CDC20 and is recruited by BUB1. CDC20 is phosphorylated by BUB1-Plk1 [13].

The role of BUB1 as a prognostic marker depends on the origin of the cancer. In low grade breast cancer [14] and endometrial cancer [15] frequent BUB1 expression was seen in cancers with a favorable course, whereas in ovarian [16] and invasive breast cancer [17], frequent expression of BUB1 was associated with a poor prognosis.

In a recent meta-analysis of gene expression studies of prognostically relevant gene expression across cancers BUB1 was found to be a prognostic marker specifically in GC [18]. Here, we comprehensively analyzed the protein expression pattern and prognostic role of BUB1 and Ki-67 by immunohistochemistry (IHC) using tissue microarrays (TMAs).

## RESULTS

### Clinico-pathological data

218 patients undergoing gastrectomy were included in this study (Table 1). The median age of GC patients was 70 years. 44 cases (20.2%) were categorized as UICC stage I, 94 cases (43.1%) were UICC II, 52 cases (23.9%) were UICC III and 28 cases (12.8%) were categorized as UICC IV stage, according to UICC TNM Classification of Malignant Tumors, seventh edition [19]. All cases were classified according to the Laurén classification and current WHO classification [20]. According to the classification of Laurén 112 cases were (51.4%) of the intestinal type, 82 (37.6%) diffuse type, 18 (8.3%) mixed type and six (2.7%) intermediate type carcinomas. 17 cases (7.8%) were categorized as pT1, 75 cases (34.4%) as pT2, 103 cases (47.2%) as pT3 and 23 cases (10.6%) were categorized as pT4. 61 cases (28.0%) had no lymph node metastasis (pN0), 79 cases (36.2%) had one or two lymph node metastases, 52 cases (23.9%) had three to six lymph node metastases and 26 cases (11.9%) had more than seven lymph node metastases.

**Table 1:** Clinico-pathologic data of 218 cases of gastric adenocarcinoma and correlation with BUB1 and Ki-67 expression (non-parametric Spearman rank test)

	Total		BUB1 expression (%)		Ki-67 expression (%)	
	n	(%)	r	*p*	r	*p*
**Gender**	218		0.052	0.448	0.121	0.082
Female	69	(31.7)				
Male	149	(68.3)				
**Age**	215		-0.047	0.493	-0.096	0.171
<40	5	(2.3)				
40-<50	15	(7.0)				
50-60	24	(11.2)				
60-<70	60	(27.9)				
70-<80	84	(38.5)				
>80	27	(12.4)				
**Tumor stage (T)**	218		-0.295	**<0.001**	-0.165	**0.017**
T1 (a/b)	17	(7.8)				
T2	75	(34.4)				
T3	103	(47.2)				
T4 (a/b)	23	(10.6)				
**Lymph node metastasis (N)**	218		-0.150	**0.027**	-0.090	0.196
N0	61	(28.0)				
N1	79	(36.2)				
N2	52	(23.9)				
N3 (a/b)	26	(11.9)				
**Distant metastasis (M)**	218		-0.184	**0.006**	-0.086	0.218
M0	190	(87.2)				
M1	28	(12.8)				
**Lymphatic vessel invasion (L)**	217		-0.062	0.362	0.037	0.597
L0	140	(64.5)				
L1	77	(35.5)				
**Vascular invasion (V)**	218		-0.100	0.140	0.004	0.959
V0	202	(92.7)				
V1	16	(7.3)				
**Residual tumor (R)**	217		-0.141	**0.038**	-0.155	**0.026**
R0	184	(84.8)				
R1	31	(14.3)				
R2	2	(0.9)				
**Histologic grade (G)**	218		0.031	0.646	0.019	0.787
G1	10	(4.6)				
G2	54	(24.8)				
G3	153	(70.2)				
G4	1	(0.5)				
**UICC stage**	218		-0.316	**<0.001**	-0.190	**0.006**
I	44	(20.2)				
II	94	(43.1)				
III	52	(23.9)				
IV	28	(12.8)				

#### BUB1 expression in gastric adenocarcinoma

**Figure 1:**
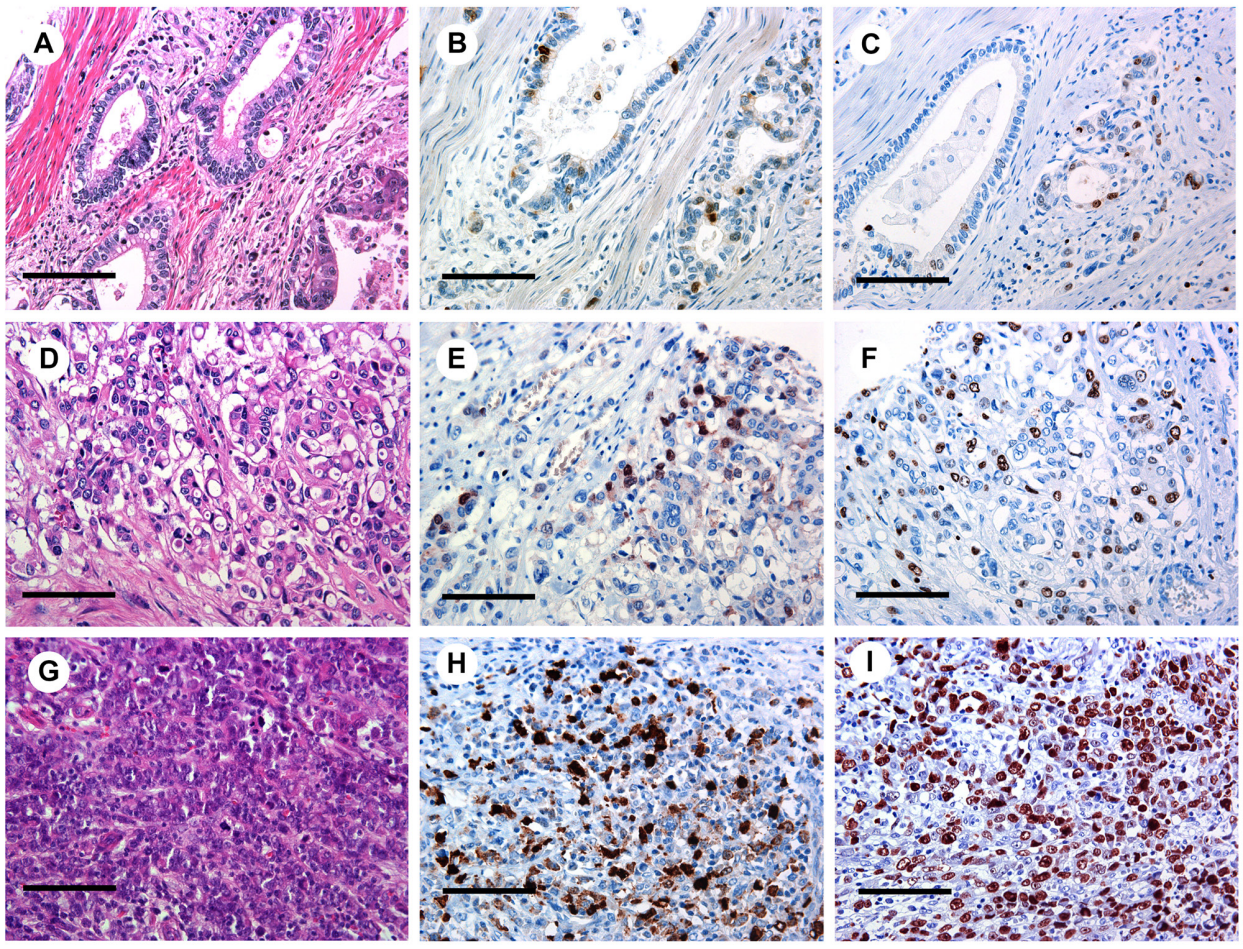
BUB1 and Ki-67 expression in gastric adenocarcinoma Low **(B)**, medium **(E)** and high **(H)** BUB1 staining with corresponding H&E **(A, D, G)** and Ki-67 **(C, F, I)** IHC staining (original magnification x400) in gastric cancer. Scale bar representing 100 μm.

The number of tumor nuclei expressing BUB1 was measured. We divided cases with different frequency of BUB1 expression into “low, medium and high” based on quartiles with low expression representing any staining frequency below the 25th percentile (8% of tumor nuclei) and high expression above the 75th percentile (28% of tumor nuclei). Out of 218 GC cases, high BUB1 expression was detected in 53 cases (24.3%), medium expression in 107 (49.1%) of cases and low BUB1 expression was seen in 58 (26.6%) cases. As depicted in Figure 1, anti-BUB1 immunohistochemical staining was found mostly in a nuclear pattern. However, in a small subset of cases, especially in well differentiated tubular forms of GC, weak cytoplasmic expression in some tumor cells was observed in addition to nuclear staining. Frequency of BUB1 expressing tumor nuclei ranged from 0.5% and 81% of all neoplastic cells (median 16%).

#### Association of BUB1 with clinico-pathological data

By non-parametric Spearman rank correlation analysis, the frequency of BUB1 expression showed a significant correlation with tumor stage (p < 0.001, r = -0.295), nodal status (p = 0.027, r =-0.15), the presence of distant metastases (p = 0.006, r =-0.184), UICC stage (p < 0.001, r = -0.316) and resection margin (p = 0.038, r = -0.141) (Table 1). There was no significant correlation between BUB1 and histological subtype, gender, patients’ age, lymphatic vessel invasion, vascular invasion or grade.

#### Ki-67 expression in gastric adenocarcinoma and association of Ki-67 with clinico-pathologic data

Tumor cells expressing nuclear Ki-67 were quantified. Based on the median, Ki-67 expression was divided into low (<33%) and high frequency (>33%) of expression. 5 out of 206 cases showed Ki-67 expression in less than 1% of tumor nuclei. The median Ki-67 protein expression was 33% with a range between 0% and 85%. 104 out of 206 (50.5%) cases showed high expression (>33% of tumor nuclei) and low expression (<33% of tumor nuclei) was detected in 102 out of 206 (49.5%) cases.

By non-parametric Spearman rank correlation analysis, Ki-67 expression was significantly associated with early tumor stage (p = 0.017, r = -0.165), complete resection (R0, p = 0.026, r =-0.155) and low UICC stage (p = 0.011, r = -0.177) (Table 1). There was also a significant correlation between Ki-67 and Laurén classification with lower Ki-67 expression often observed in diffuse type and higher Ki-67 expression in intestinal type of GC (p = 0.002) (Table 2). Ki-67 did not correlate with other clinico-pathologic parameters (gender, age at disease onset, the presence of distant metastases, lymphatic spread, vascular invasion or histological grade).

**Table 2:** Ki-67 correlates with histological subtype according to Laurén classification in gastric adenocarcinoma (Fisher's exact test)

	Total	Dichotomized Ki-67				Ki-67 split into quartiles				
			Ki-67 low	Ki-67 high		Ki-67 first	Ki-67 second	Ki-67 third	Ki-67 fourth	
	n	(%)	[n (%)]	[n (%)]	*p*	quartile [n (%)]	quartile [n (%)]	quartile [n (%)]	quartile [n (%)]	*p*
**Laurén**	218				0.002					0.010
Diffuse	81	(37.2)	48 (22.0)	27 (12.4)		28 (12.8)	20 (9.2)	15 (6.9)	12 (5.5)	
Intestinal	113	(51.8)	42 (19.3)	67 (30.7)		18 (8.3)	24 (11.0)	35 (16.1)	32 (14.7)	
Mixed	18	(8.3)	11 (5.0)	5 (2.3)		5 (2.3)	6 (2.8)	3 (1.4)	2 (0.9)	
Intermediate	6	(2.8)	3 (1.4)	3 (1.4)		2 (0.9)	1 (0.5)	0 (0.0)	3 (1.4)	

#### Correlation of BUB1 and Ki-67 protein expression

**Figure 2:**
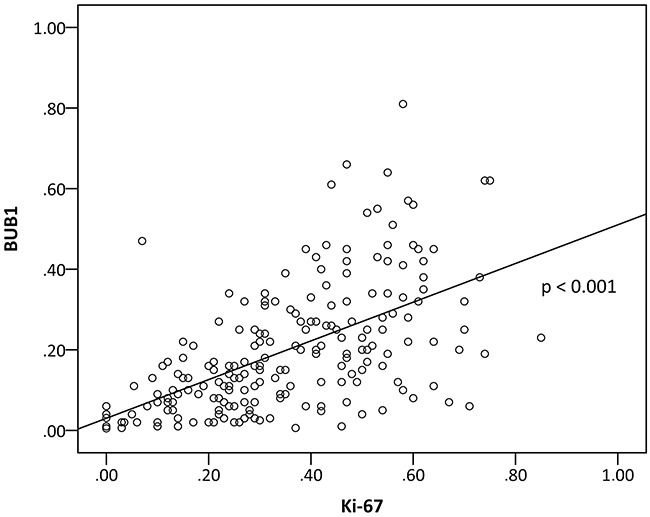
Correlation of BUB1 and Ki-67 expression (r = 0.580, p < 0.001, Spearman rank test).

BUB1 and Ki-67 expression were directly correlated (p < 0.001, r = 0.580) in GCs (Figure 2).

### Overall survival analysis

Clinical follow-up data was available for 148 cases (67.9%) with an estimated median overall survival (OS) of 25 months.

Kaplan-Meier analysis confirmed that tumor stage (p = 0.014), nodal status (p < 0.001), lymphangiosis carcinomatosa (p = 0.025), hemangiosis carcinomatosa (p = 0.017), positive resection margin (p < 0.001), grade (p = 0.041) and UICC stage (p = 0.007) correlated inversely with OS, and Ki-67 correlated directly with OS (p = 0.001). Furthermore, intestinal type and low grade (G1, G2) GC showed a longer OS when compared to diffuse type and high grade (G3, G4) GC (p = 0.036 and p = 0.031, respectively).

By Kaplan-Meier analysis BUB1 expression was examined in two independent cohorts of GC. Low BUB1 expression was significantly associated with an adverse prognosis in the first cohort (n = 119) (log-rank test, p = 0.002) and in the second cohort (n = 99) (log-rank test, p < 0.001) (see Supplementary Materials, Supplementary Figure 1).

**Figure 3:**
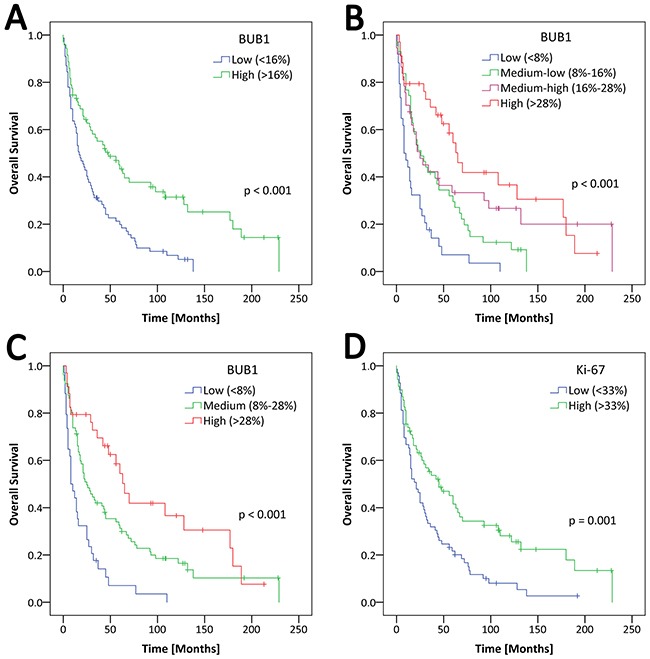
Overall survival in patients with gastric adenocarcinoma is dependent on BUB1 expression Kaplan-Meier analysis with follow-up data available for 148 out of 218 cases. **(A)** Two-tier BUB1 analysis. Low BUB1 expression (<16% of tumor nuclei) (blue curve) with a median OS of 17 months (95% CI, 10.747-23.254 months), high BUB1 expression (>16% of tumor nuclei) (green curve) with an estimated median survival of 48 months (95% CI, 23.445-72.555 months) (p < 0.001, log-rank test). **(B)** Four-tier BUB1 analysis. Low BUB1 expression (<8%) (blue curve) with a median OS of 8 months (95% CI, 3.238-12.762 months), medium-low BUB1 expression (8%-16%) (green curve) with a median OS of 26 months (95% CI, 13.151-38.849 months), medium-high expression (16%-28%) (purple curve) with a median OS of 25 months (95% CI, 9.577-40.423 months), high BUB1 expression (>28%) (red curve) with an estimated median survival of 65 months (95% CI, 49.375-80.625 months) (p < 0.001, log-rank test). **(C)** Three-tier BUB1 analysis. Low BUB1 expression (<8%) (blue curve) with a median OS of 8 months (95% CI, 3.238-12.762 months), medium BUB1 expression (8%-28%) (green curve) with a median OS of 25 months (95% CI, 15.555-34.445 months), high expression (>28%) (red curve) with an estimated median survival of 65 months (95% CI, 49.375-80.625 months) (p < 0.001, log-rank test). **(D)** Two-tier Ki-67 analysis. Low BUB1 expression (<33%) (blue curve) with a median OS of 21 months (95% CI, 12.867-29.133 months), high BUB1 expression (>33%) (green curve) with an estimated median survival of 45 months (95% CI, 22.000-68.000 months) (p = 0.001, log-rank test).

When the data of both cohorts was tested by Kaplan-Meier analysis significant differences in OS within the low, medium and high BUB1 expression group (p < 0.001) (Figure 3C) were found using a three-tier system. Patients with high BUB1 expression (n = 34) had a median OS of 65 months (95% CI, 49.375-80.625 months), the median OS within the medium BUB1 expression group (n = 80) was 25 months (95% CI, 15.555-34.445months) and the low BUB1 expression group (n = 34) had a median OS of only 8 months (95% CI, 3.238-12.762 months) (log-rank test, p < 0.001).

The three-tier system (Figure 3C) showed the best separation in prognostic subgroups by BUB1 expression, when compared to a two-tier separation (Figure 3A) or a four-tier system based on quartiles (Figure 3B). Therefore, the three-tier system was used for Kaplan-Meier analysis.

14 out of 128 patients with GC were treated with neoadjuvant chemotherapy. Interestingly, high BUB1 expression in tumors of patients receiving neoadjuvant chemotherapy (>28% of tumor nuclei) was associated with a shorter overall survival by Kaplan-Meier analysis compared to patients with tumors with BUB1 expression in less than 28% of tumor nuclei (log-rank test, p = 0.028).

For univariate and multivariate comparative analysis binary variables were used, as follows (Table 3).

**Table 3:** Univariate and multivariate analysis of clinico-pathologic parameters and overall survival in gastric cancer patients

Parameter	Category	Overall survival		*p*
		HR	95% CI	
Univariate				
Tumor stage pT	pT1/pT2 vs. pT3/pT4	1.683	1.166-2.429	**0.005**
Nodal status, pN	pN0 vs. pN1/pN2/pN3	1.970	1.306-2.972	**0.001**
Metastasis status, pM	pM0 vs. pM1	1.301	0.765-2.211	0.331
Lymphatic vessel invasion, L	L0 vs. L1	1.535	1.047-2.250	0.028
Vascular invasion, V	V0 vs. V1	2.238	1.124-4.456	0.022
Residual tumor, R	R0 vs. R1/R2	1.691	1.006-2.845	0.048
Histologic grade, G	G1/G2 vs. G3/G4	1.539	1.034-2.292	0.034
UICC stage	I/II vs. III/IV	1.729	1.183-2.528	**0.005**
BUB1 expression	low vs. high	0.476	0.328-0.692	**<0.001**
Ki-67 expression	low vs. high	0.546	0.374-0.797	**0.002**
Laurén classification	intestinal vs. diffuse	0.665	0.451-0.981	0.040
Multivariate				
Tumor stage pT	pT1/pT2 vs. pT3/pT4	1.312	0.885-1.944	0.177
Nodal status, pN	pN0 vs. pN1/pN2/pN3	1.669	1.074-2.594	**0.023**
UICC stage	I/II vs. III/IV	1.209	0.765-1.909	0.417
BUB1 expression	low vs. high	0.604	0.394-0.927	**0.021**
Ki-67 expression	low vs. high	0.699	0.464-1.053	0.086

Univariate Cox regression survival analysis showed that tumor stage, pT (p = 0.005), nodal status, pN (p = 0.001), lymphangiosis carcinomatosa (p = 0.028), hemangiosis carcinomatosa (p = 0.022), resection margin (p = 0.048), histological grade (p = 0.034), UICC stage (p = 0.005), Laurén classification (p = 0.04), Ki-67 expression (p = 0.002) and BUB1 expression (p < 0.001) are all associated with OS.

Multivariate Cox regression analysis confirmed nodal status (p = 0.023) and BUB1 expression (p = 0.021) as independent prognostic markers.

By multivariate Cox regression analysis we further analyzed the prognostic value of all parameters which had a p < 0.01 by univariate survival analysis (tumor stage, nodal status, UICC stage, Ki-67 expression and BUB1 expression). Multivariate Cox regression analysis confirmed that BUB1 expression (p = 0.021) and pN category (p = 0.023) were the most significant and independent prognostic factors for OS in this cohort of GC (Table 3).

## DISCUSSION

In our cohort of 218 patients with GC we can demonstrate that frequent BUB1 expression in tumor nuclei is associated with a good prognosis in patients with GC, and that BUB1 in only a small fraction of tumor cells is associated with a poor prognosis. BUB1 seems to be an independent prognostic marker in GC patients. Our results extend and confirm previous findings that demonstrated upregulation of all members of the BUB gene family (BUB1, BUBR1, and Bub3) at the mRNA level in GC [10].

In this study we show a highly significant correlation with OS, rendering BUB1 a potentially useful clinical prognostic marker.

Cell cycle associated proteins are frequently aberrantly expressed in cancer [16] and can be targeted using small molecular inhibitory compounds such as novel inhibitors of the BUB1-Polo-like-kinase 1 interaction [21] and other cell cycle kinase inhibitors currently tested in preclinical studies [22]. Transcriptomic in silico analysis of gene expression of mitotic components revealed overexpression of five kinases including BUB1, TTK protein kinase, Citron Rho-interacting kinase (CIT), ZAK and NEK2 in GC [23]. Because SAC proteins Mad2 and BUBR1 have been shown to be involved in GC tumor progression, treatment of GC with Mad2 and BUBR1 inhibitors has been proposed as a novel treatment modality [24]. In the future, assessment of BUB1 expression in GC tissues may be useful in predicting the response to cell cycle kinase inhibitors in clinical trials using anti-proliferative and/or checkpoint inhibitors.

Our finding that high proliferative rate defined by Ki-67 staining correlates with a good prognosis confirms a recent meta-analysis showing different correlations of Ki-67 with survival in patients with GC, which may be due to analytical methods and/or patient characteristics [8]. Other proliferation markers such as PCNA (proliferating cell nuclear antigen) and MCM family members (mini-chromosome maintenance) have been examined, however, no prognostic value could be demonstrated [25].

BUB1 is a cell cycle protein with kinase function and part of the SAC [26]. Few studies have examined BUB1 in human cancer so far. In endometrial carcinoma [15] and low-grade breast cancer [14] high frequency of BUB1 is associated with a good prognosis while in invasive breast cancers [17] and ovarian cancer [16] BUB1 is associated with a poor prognosis. In colon carcinomas reduced BUB1 mRNA levels were associated with shorter relapse-free survival after surgery [27].

One reason for the different roles of BUB1 in different types of cancers may be because of differences in expression level: BUB1 promotes cell death in response to chromosomal missegregation and acts to suppress spontaneous tumorigenesis in knockout and hypomorphic mouse model systems population [28]. Thus, a certain level of BUB1 may be important in order to prevent tumorigenesis. However, when BUB1 is overexpressed in transgenic mouse models it drives tumorigenesis and aneuploidy and excessive BUB1 may have an adverse effect by causing genetic instability [29].

GCs are frequently aneuploid [30] and BUB1 has been shown not to be mutated in GC [31]. A simple mechanistic explanation, such as that BUB1 expression might confer protection against aneupleudity due to missegregation of chromosomes during metaphase seems unlikely in the light of earlier findings of Grabsch et al. [11], who could not show a correlation of BUB1 expression and microsatellite stability or DNA ploidity in GC.

BUB1 acts as a scaffold at kinetochores to recruit Plk1 and all components of the MCC [13]. BUB1 and Plk1 represent parallel, but not redundant mechanisms to inhibit the APC/C. The cell division cycle 20 homolog (CDC20) is increased in poor prognostic GC [32] and is a target molecule in the cell cycle checkpoint that activates the APC/C. Thus it is possible, that CDC20 phosphorylation induced by BUB1 leads to checkpoint-dependent mitotic arrest in good prognostic GC. Clearly, additional *in vitro* studies using GC cell lines are necessary to investigate these hypotheses further.

A difference of 57 months for the median OS between the low and high BUB1 expression groups (p < 0.001) strongly indicates that BUB1 as a prognostic marker may be a clinically relevant finding (Figure 3). In addition to poor survival, patients with GC showing low BUB1 expression had a significantly higher tumor stage (p < 0.001), higher rates of lymph node metastases (p = 0.027) and distant metastases (p = 0.006) and a significantly higher UICC stage (p < 0.001), which may be another reason for the poor survival in this group and may suggest closer clinical follow up of patients, whose tumors show low BUB1 expression.

In patients treated with neoadjuvant chemotherapy high BUB1 expression is associated with a poor prognosis. This result is plausible, as chemotherapy in the neoadjuvant setting targets the proliferative capacity of tumor cells and those tumors with high proliferative rates post chemotherapy at the time of resection do not appear to have profited from the neoadjuvant treatment. Obviously, this observation warrants further study in a larger patient cohort or prospective clinical trial.

Finally, cell cycle aberrations are a hallmark of cancer and targeting mitotic checkpoints while showing promising pre-clinical results are hampered by serious side effects. Dependence on BUB1 expression for mitotic arrest and suppression of BUB1 in poor prognostic GC may reveal novel strategies to refine cell cycle targeted therapeutics [33].

We suggest integrating BUB1 protein expression into risk stratification protocols with low BUB1 expression representing any frequency of nuclear expression less than 8%, medium expression between 8% and 28% and high expression above 28%. The exact role of BUB1 in GC will need to be examined by further prospective clinical studies as well as functional assays *in vitro*.

## MATERIALS AND METHODS

### Patient cohort

Tissue samples of two hundred eighteen (218) patients with GC who underwent surgery between 1994 and 2013 were enclosed in this study. All samples were retrieved from the Department of Pathology, University Hospital Bonn, in accordance with the local ethics committee. Clinico-pathological information for 218 GC cases was collected by reviewing clinical and pathologic records. Clinical follow-up data was available for 148 patients of our cohort (67.9%). Median follow up time of all cases was 25 months. There were 122 events (82.4%) and 26 cases (17.6%) were censored.

### Construction of TMAs

TMA construction was performed as described earlier [34]. Briefly, formalin-fixed, paraffin-embedded tissue samples were used for constructing TMAs. Three 0.5mm cores from the tumor-containing donor blocks were inserted into a recipient paraffin block. To represent carcinomatous tissue sufficiently, fixed paraffin blocks containing different areas of the tumor (intramucosal, lamina propria, and muscularis propria invasion) were used for sampling, and three different areas containing dense tumor areas were chosen for punch biopsies.

### Immunohistochemistry

TMA and standard paraffin sections (2-3 μm) were placed in 200 mL of target retrieval solution (citrate buffer pH 6.0) and heated for 20 minutes at boiling temperature. Afterwards, sections were washed with Tris-buffered saline. Immunohistochemical staining for Ki-67 was performed with a semi-automatic immunohistochemistry stainer (Autostainer 480; Medac, Germany) using the horse radish-peroxidase polymer method. Endogenous peroxidase activity was blocked by treatment with H_2_0_2_ for ten minutes. For BUB1 staining slides were developed with OptiView DAB IHC Detection Kit (Ventana Medical Systems). Tissue sections were stained with hematoxylin and eosin (H&E). For immunohistochemical (IHC) staining the following primary antibodies were used: BUB1 (rabbit monoclonal antibody, clone: EPR18947, dilution 1:100, Abcam) and Ki-67 (clone K-2, Zytomed MSK 018, dilution 1:500, Zytomed). Human testis tissue was used as external positive control and centroblasts in germinal centers as an internal positive control for both stains.

### Scoring

BUB1 and Ki-67 protein expression was quantified by using the semi-quantitative image analysis software “Tissue studio” (v.2.1), Definiens AG, Munich, Germany, as described earlier [35, 36]. First, all slides were scanned and images digitalized with a Zeiss MIRAX scanner (Carl Zeiss, Oberkochen, Germany). After manually choosing tumor areas the program was run on all TMA slides quantifying immunopositive nuclei within the region of interest (ROI). The frequency of nuclear protein expression of BUB1 and Ki-67 was calculated by determining the positive index defined as the quotient of IHC positive and all tumor nuclei regardless of staining intensity.

For immunohistochemistry at least 2 of the 3 cores per case were analyzed for BUB1 and Ki-67 staining. All TMA cores were analyzed individually for BUB1 and Ki-67 scores and an average score of all interpretable cores was calculated. A minimum of 100 tumor nuclei was counted per core and at least 800 tumor nuclei total per case.

The frequency of BUB1 and Ki-67 expression in tumor nuclei were assessed semi-quantitatively using tissue microarrays and confirmed using representative standard paraffin sections in selected cases by D.S. and two independent pathologists (I.G. and M.B.) who were blinded to clinico-pathological data.

The two-, three- and four-tier scoring systems are explained in the results part.

### Statistical analysis

Statistical analysis was performed using the software package IBM SPSS (Chicago, IL) Statistics for Windows (version 24). For comparison of BUB1 and Ki-67 protein expression with clinico-pathologic parameters (gender, age, tumor stage, nodal status, metastasis status, lymphatic vessel invasion, vein invasion, resection status, grade and UICC stage) a non-parametric Spearman's rank correlation coefficient was calculated. The relation between Ki-67 expression and histologic type of GC was evaluated by Fisher's exact test. The correlation between BUB1 and Ki-67 expression was examined using Spearman's rank correlation coefficient. OS estimates were calculated according to the Kaplan-Meier method using log-rank test. Univariate and multivariate analysis were performed using Cox regression. Multivariate Cox regression analysis was run backwards with p(in) = 0.05 and p(out) = 0.1. Hazard ratios (HR) and their 95% confidence intervals (95% CI) were calculated. All tests were 2-sided, and p values <0.05 were considered statistically significant.

## SUPPLEMENTARY MATERIALS FIGURES AND TABLES



## Abbreviations

GC: Gastric adenocarcinoma
BUB1: budding uninhibited by benzimidazoles 1
SAC: spindle assembly checkpoint
APC/C: anaphase promoting complex or cyclosome
MCC: mitotic checkpoint complex
IHC: immunohistochemistry
ROI: region of interest
CDC20: cell division cycle 20 homolog
