# Supplementary material for: Low BUB1 expression is an adverse prognostic marker in gastric adenocarcinoma
Source: Oncotarget. 2017 Jul 18;8(44):76329–39. doi: 10.18632/oncotarget.19357 (PMC5652709; doi:10.18632/oncotarget.19357)
Supplement: Supplementary file 1 [file oncotarget-08-76329-s001.pdf]

Low BUB1 expression is an adverse prognostic marker in gastric adenocarcinoma

SUPPLEMENTARY MATERIALS

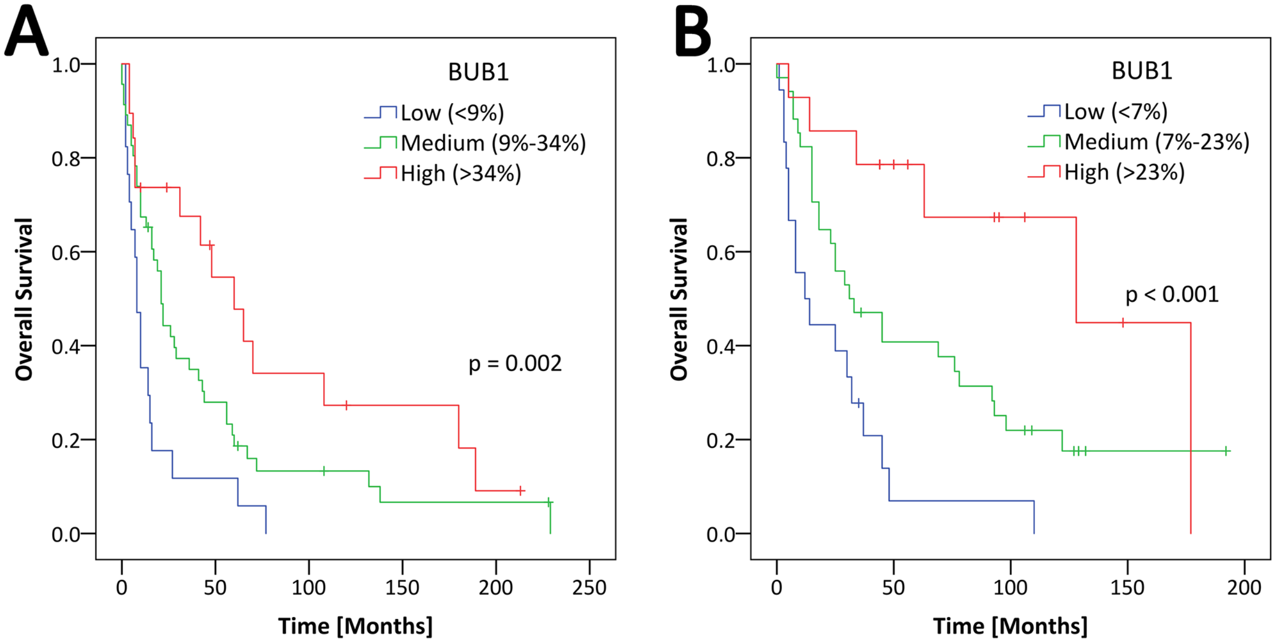

Supplementary Figure 1: BUB1 expression in two independent cohorts of patients with gastric adenocarcinoma. Kaplan-Meier analysis with follow-up data was available for 82 out of 119 cases in the first cohort and 66 out of 99 cases in the second cohort. **(A)** Three-tier BUB1 analysis of the first cohort. The frequency of BUB1 expression was subdivided into "low, medium and high" with low expression representing any staining frequency below the 25th percentile (9% of tumor nuclei) and high expression above the 75th percentile (34% of tumor nuclei). Low BUB1 expression (<9%) (blue curve) with a median OS of 8 months (95% CI, 4.975-11.025 months), medium BUB1 expression (9%-34%) (green curve) with a median OS of 21 months (95% CI, 17.214-24.786 months), high expression (>34%) (red curve) with an estimated median survival of 60 months (95% CI, 32.731-87.269 months) ($p = 0.002$, log-rank test). **(B)** Three-tier BUB1 analysis of the second cohort. The frequency of BUB1 expression was subdivided into "low, medium and high" with low expression representing any staining frequency below the 25th percentile (7% of tumor nuclei) and high expression above the 75th percentile (23% of tumor nuclei). Low BUB1 expression (<7%) (blue curve) with a median OS of 12 months (95% CI, 0.000-24.473 months), medium BUB1 expression (7%-23%) (green curve) with a median OS of 31 months (95% CI, 8.736-53.264 months), high expression (>23%) (red curve) with an estimated median survival of 128 months (95% CI, 11.248-244.752 months) ($p < 0.001$, log-rank test).
